# Supercritical carbon dioxide decellularization of plant material to generate 3D biocompatible scaffolds

**DOI:** 10.1038/s41598-021-83250-9

**Published:** 2021-02-11

**Authors:** Ashlee F. Harris, Jerome Lacombe, Sumedha Liyanage, Margaret Y. Han, Emily Wallace, Sophia Karsunky, Noureddine Abidi, Frederic Zenhausern

**Affiliations:** 1 Center for Applied NanoBioscience and Medicine, College of Medicine Phoenix, University of Arizona, 475 North 5th Street, Phoenix, AZ 85004 USA; 2 Department of Basic Medical Sciences, College of Medicine Phoenix, University of Arizona, 475 N 5th Street, Phoenix, AZ 85004 USA; 3 Fiber and Biopolymer Research Institute, Department of Plant and Soil Science, Texas Tech University, Lubbock, TX USA; 4 School of Pharmaceutical Sciences, University of Geneva, Geneva, Switzerland; 5 University of Arizona COM – Phoenix, Biomedical Sciences Partnership Building, 6th Floor, 475 North 5th Street, Phoenix, AZ 85258 USA

**Keywords:** Biomedical materials, Biomedical engineering

## Abstract

The use of plant-based biomaterials for tissue engineering has recently generated interest as plant decellularization produces biocompatible scaffolds which can be repopulated with human cells. The predominant approach for vegetal decellularization remains serial chemical processing. However, this technique is time-consuming and requires harsh compounds which damage the resulting scaffolds. The current study presents an alternative solution using supercritical carbon dioxide (scCO_2_). Protocols testing various solvents were assessed and results found that scCO_2_ in combination with 2% peracetic acid decellularized plant material in less than 4 h, while preserving plant microarchitecture and branching vascular network. The biophysical and biochemical cues of the scCO_2_ decellularized spinach leaf scaffolds were then compared to chemically generated scaffolds. Data showed that the scaffolds had a similar Young’s modulus, suggesting identical stiffness, and revealed that they contained the same elements, yet displayed disparate biochemical signatures as assessed by Fourier-transform infrared spectroscopy (FTIR). Finally, human fibroblast cells seeded on the spinach leaf surface were attached and alive after 14 days, demonstrating the biocompatibility of the scCO_2_ decellularized scaffolds. Thus, scCO_2_ was found to be an efficient method for plant material decellularization, scaffold structure preservation and recellularization with human cells, while performed in less time (36 h) than the standard chemical approach (170 h).

## Introduction

As the need for replacement organs and tissues increases, the field of tissue engineering has risen to meet this challenge by seeking original sources of biomaterial and alternative approaches for their generation^1^. In this perspective, scaffolds mimicking the in vivo tissue environment are sought to provide appropriate structural and biomechanical support to cells while simultaneously facilitating cell behavior and tissue development^2–5^. The recent emergence of plant-based materials offers a promising opportunity to meet this need^6–9^.

Indeed, several studies have found that vegetal material could be decellularized to generate biocompatible scaffolds^6–16^. Plants are a natural, renewable material source which are easily accessible and offer a low-cost alternative to animal tissue^17,18^. They are comprised primarily of cellulose, a biocompatible compound that has already been extensively used in the medical industry to dress wounds or create artificial skin^18–24^. Due to its inherent strength, porosity and water-retention properties, cellulose has been demonstrated as a viable choice from which to generate biomaterials^25^. Another advantage of plant-based biomaterials is their unique intrinsic structures, which offer a wide variety of microscale patterned constructs resembling the complex cellular microenvironment^15,17^. Moreover, the natural branching fluid transport system found in plants is similar to a mammalian blood vessel network, with detailed definition and small-scale vessels that cannot be simply reproduced by the current 3D printers or microfluidic technologies^26^.

Upon re-cellularization, it has been demonstrated that human cells can attach, metabolize, proliferate and align to the surface microtopography of plant scaffolds^9,13,27^ while the inner plant vasculature can also be endothelialized^8,10^. Interestingly, plant scaffolds have been tested in vivo and found to be pro-angiogenic, where blood vessels grew throughout the biomaterial demonstrating that the scaffold in vivo generates a low inflammatory response, while promoting cell invasion and extracellular matrix (ECM) deposition^7^. In addition, the stiffness of decellularized plant tissue has also been shown to match the stiffness of specific human anatomical tissue sites^11^.

The gold standard method to decellularize plant tissue is serial chemical treatment. Traditionally, an aqueous detergent (e.g. Sodium Dodecyl Sulfate (SDS)) is followed with a surfactant-bleach solution. While this approach has been effective, the scaffold preparation takes several days. The scaffolds must be washed numerous times to ensure harsh chemicals are removed and not depositing a toxic residue^15,28^. Moreover, in animal tissue, these chemicals have been shown to damage the scaffold during decellularization^4,28–30^. For example, the use of SDS, a powerful cell lysing agent which disrupts protein configuration and native ultrastructure, has been shown to induce collagen compaction in decellularized heart valves or ECM fibrosis in lungs^31–34^. This type of disruption can affect cell signaling, alignment and organization upon recellularization. While the effects on plant tissue are unexplored, novel processing methods are needed to perform efficient decellularization with shorter treatment times and without the use of harsh chemicals.

Supercritical carbon dioxide (scCO_2_) offers an alternative and promising decellularization approach. In its supercritical phase, CO_2_ requires high pressure yet low temperature. Once the fluid state is achieved, scCO_2_ displays low viscosity and high diffusivity. With these gas-like transport properties, liquid-like density, and lack of surface tension, compressed carbon dioxide can penetrate dense material and act as a powerful solvent^28,35,36^. It is a green, non-toxic, low cost, and readily available technology with a low critical point (7.38 MPa, 31.1 °C) which is compatible with delicate biological tissue^36^. Thus, multiple studies looking for an alternative to chemical and enzymatic processes, recently showed that mammalian tissues can be successfully decellularized with scCO_2_, providing ECM scaffolds with improved mechanical properties that could be used to promote cell growth and angiogenesis^28,37–41^. In addition, scCO_2_ decellularization is a time-efficient approach, tissues were processed in a matter of hours and simultaneously sterilized^2^.

Therefore, we hypothesized that the scCO_2_ approach for decellularizing plant tissue could be a promising alternative to the standard method of chemical processing. Herein, we developed and evaluated the efficiency of scCO_2_ decellularization protocols on baby spinach leaves. We assessed the biochemical and biophysical properties of the resulting scaffolds and compared our results to those of chemically treated leaves. Finally, normal human skin cells were seeded on the scCO_2_ decellularized scaffolds to investigate biocompatibility.

## Results

### Effectiveness of spinach leaf decellularization by scCO_2_

**Figure 1:**
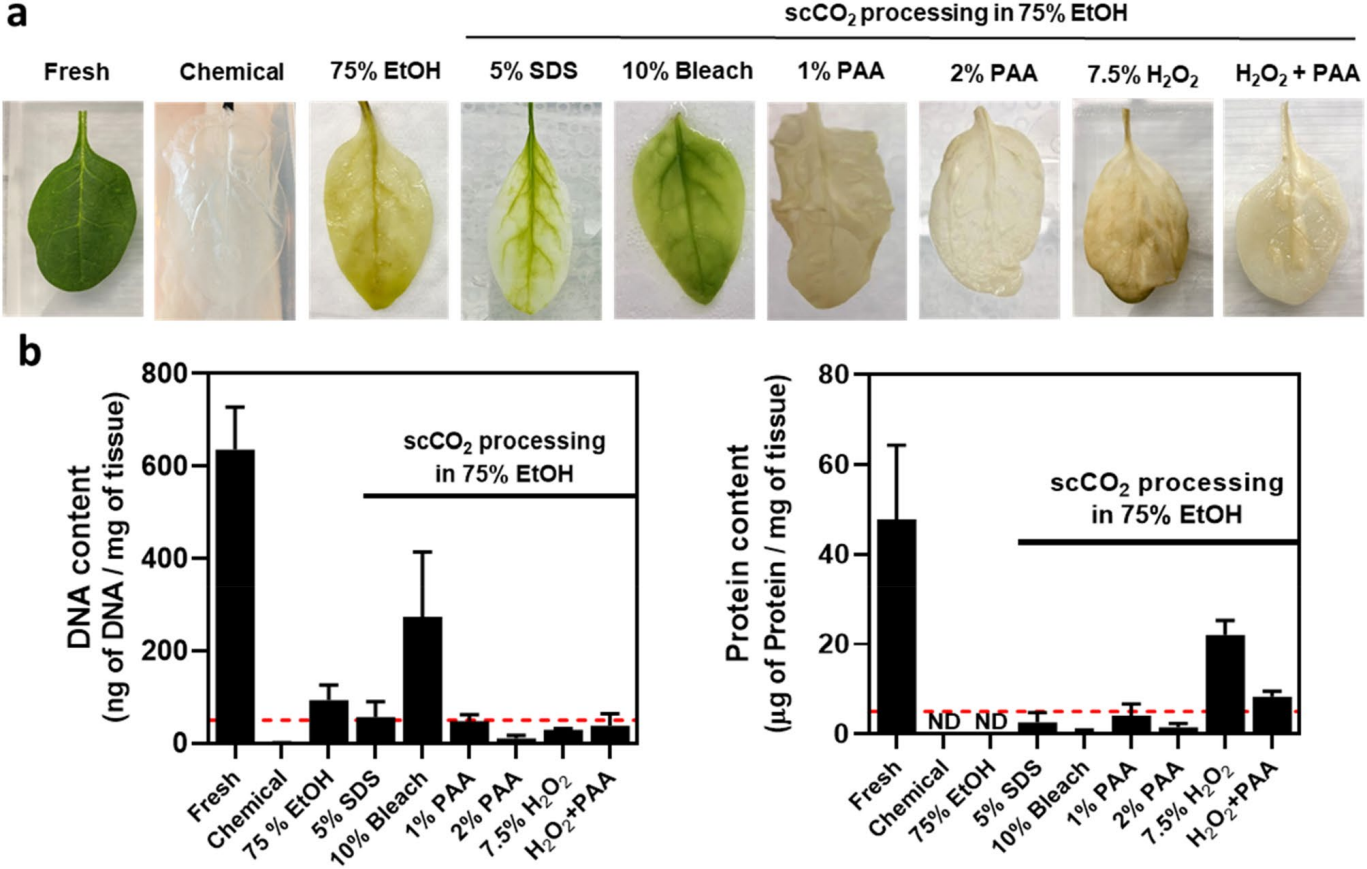
scCO_2_ decellularization of spinach leaf. (**a**) Images of fresh, chemically treated and scCO_2_ treated baby spinach leaves. scCO_2_ processing was investigated with various co-solvents. (**b**) DNA and protein content of fresh, chemical treated and scCO_2_ treated scaffolds. The dashed red lines indicate the decellularization threshold of 50 ng of DNA or 5 µg of protein per mg of tissue (data as mean ± SEM; n = 3).

Co-solvents have been commonly used with scCO_2_ to coax along the reaction and improve the decellularization efficiency^28,40,42^. Using scCO_2_ parameters of 17.23 MPa at 33 °C for 3 h, 75% ethanol alone as a co-solvent was first assessed for its ability to remove vegetal content from spinach leaves. Results found that the remaining plant material was still very green in color (Fig. 1a), demonstrating an incomplete decellularization and confirmed by DNA and protein content analysis (Fig. 1b). Next, we tested commonly known chemical decellularization agents, including SDS and sodium hypochlorite. While successful with plant content removal in chemical decellularization, these compounds do not dissolve in scCO_2_ and, not surprisingly, were found to be ineffective for enhancement of scCO_2_ processing (Fig. 1). Therefore, we investigated weak base co-solvents peracetic acid (PAA) and hydrogen peroxide (H_2_O_2_), both individually and in combination. These formulations were found to be visually more effective at removing plant content based on the lack of chlorophyll in the resulting scaffolds (Fig. 1a). DNA and protein content analysis further confirmed this observation (Fig. 1b). Processing with 2% PAA in 75% ethanol was the most effective co-solvent, showing 10.67 ng of DNA and 1.51 µg of protein per mg of plant tissue when compared to a fresh spinach leaf with 636.09 ng of DNA and 47.80 µg of protein per mg of plant tissue (Fig. 1b)^30^. We also applied this scCO_2_ decellularization processing to other plant tissues. Mint leaves (*Mentha x suavis)*, parsley stems (*Petroselinum crispum)* and celery stalks (*Apium graveoleans)* were each successfully decellularized suggesting widespread use of our technique for different type of vegetal material (Figure S1). As the most effective co-solvent, 2% PAA was selected to be used for all scCO_2_ investigations performed in this study. Moreover, to remove the remaining scaffold debris and generate a clear scaffold for immunofluorescent imaging, an additional decoloring step with sodium hypochlorite was performed. The colorless scaffolds were used for assessment in our biophysical, biochemical and biocompatibility investigation.

### Preservation of scCO_2_ scaffold ultra-structure and vascular network

**Figure 2:**
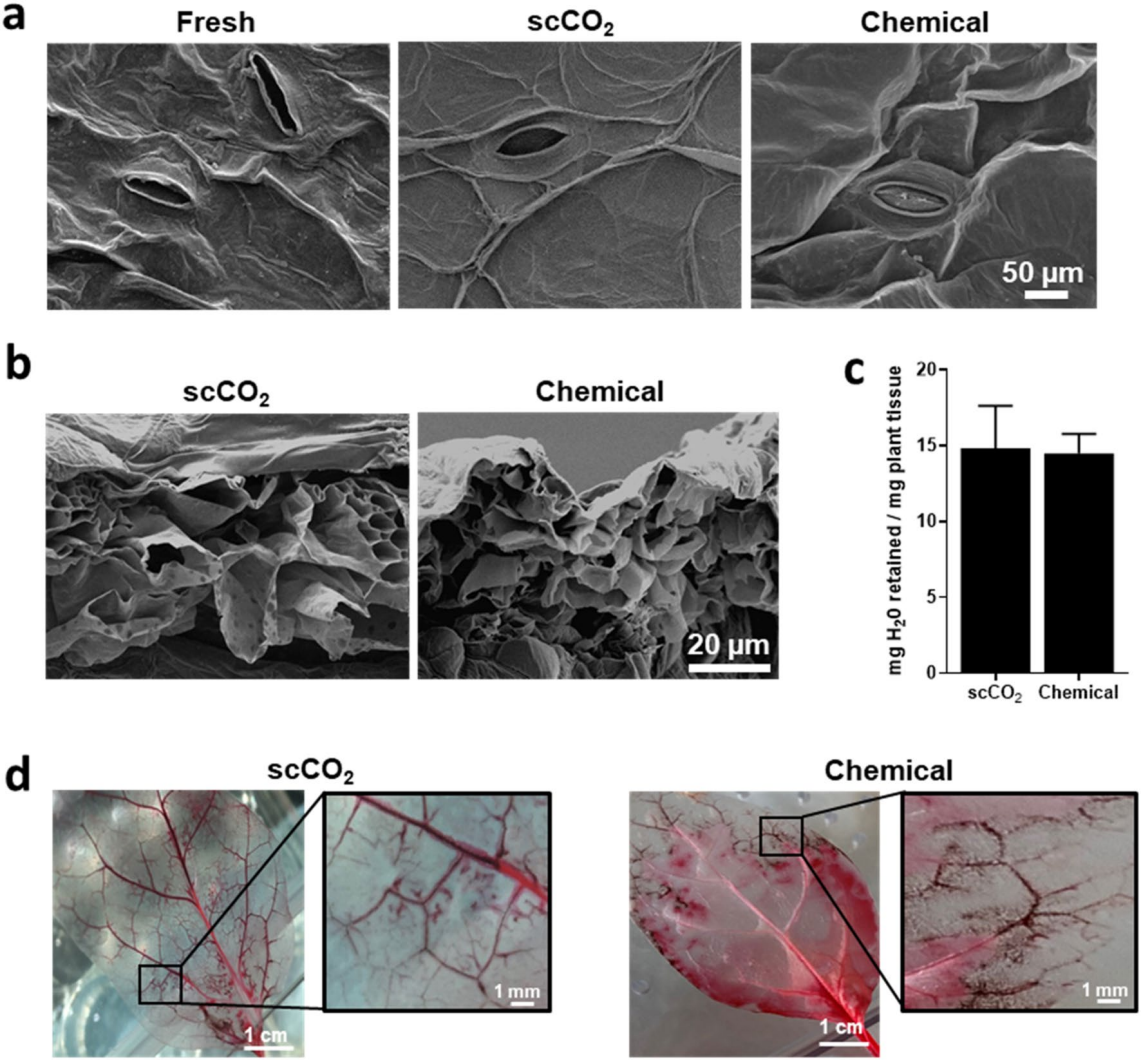
Scaffold structure after decellularization. (**a**) SEM surface images of a fresh spinach leaf, scCO_2_ and chemically decellularized scaffolds. (**b**) Cross-sectioned SEM images of scCO_2_ and chemically decellularized scaffolds. (**c**) Comparison of the mass of water (H_2_O) retained by the scCO_2_ and chemically decellularized scaffolds (n = 7). (**d**) Images of scCO_2_ and chemically decellularized scaffolds perfused with red dye showing the integrity of vascular network, down to the capillary level (inset).

Scanning electron microscopy (SEM) surface imaging revealed that the scCO_2_ decellularized leaf structures lost plant material (Fig. 2a). Epidermal plant cell walls, guard cells of the stomata and tissue of vascular bundle structures, previously hidden by the fullness of the fresh leaf, became visible. Moreover, the scCO_2_ treatment preserved the architecture and microtopographic features of plant scaffold, similar to results seen in chemically treated scaffolds (Fig. 2a). Cross-section SEM imaging further showed the extent of decellularization and the preservation of the internal porous structure with scaffold pores maintained their hierarchical shape and size, suggesting that scCO_2_ treatment may not affect the porosity (Fig. 2b). To confirm this assumption, the water retention properties of the two scaffolds were then compared (Fig. 2c) and results showed that the scCO_2_ and chemically scaffolds can indeed retain a similar amount of water (14.81 and 14.50 mg of water per mg of plant tissue respectively).

Finally, in order to assess the patency of the scCO_2_ decellularized vascular network, the vegetal scaffolds have been perfused with red dye using a capillary-evaporation method (Fig. 2d)^43^. As water evaporated from the scCO_2_ decellularized scaffold, fluid was drawn from the reservoir, reaching the main vein and traveling to even the smallest capillaries. The functional vascular network reinforces that plant architecture in scCO_2_ decellularized scaffolds is preserved, similar to chemical treatment and as previously demonstrated^8,10^.

### Stiffness assessment

**Figure 3:**
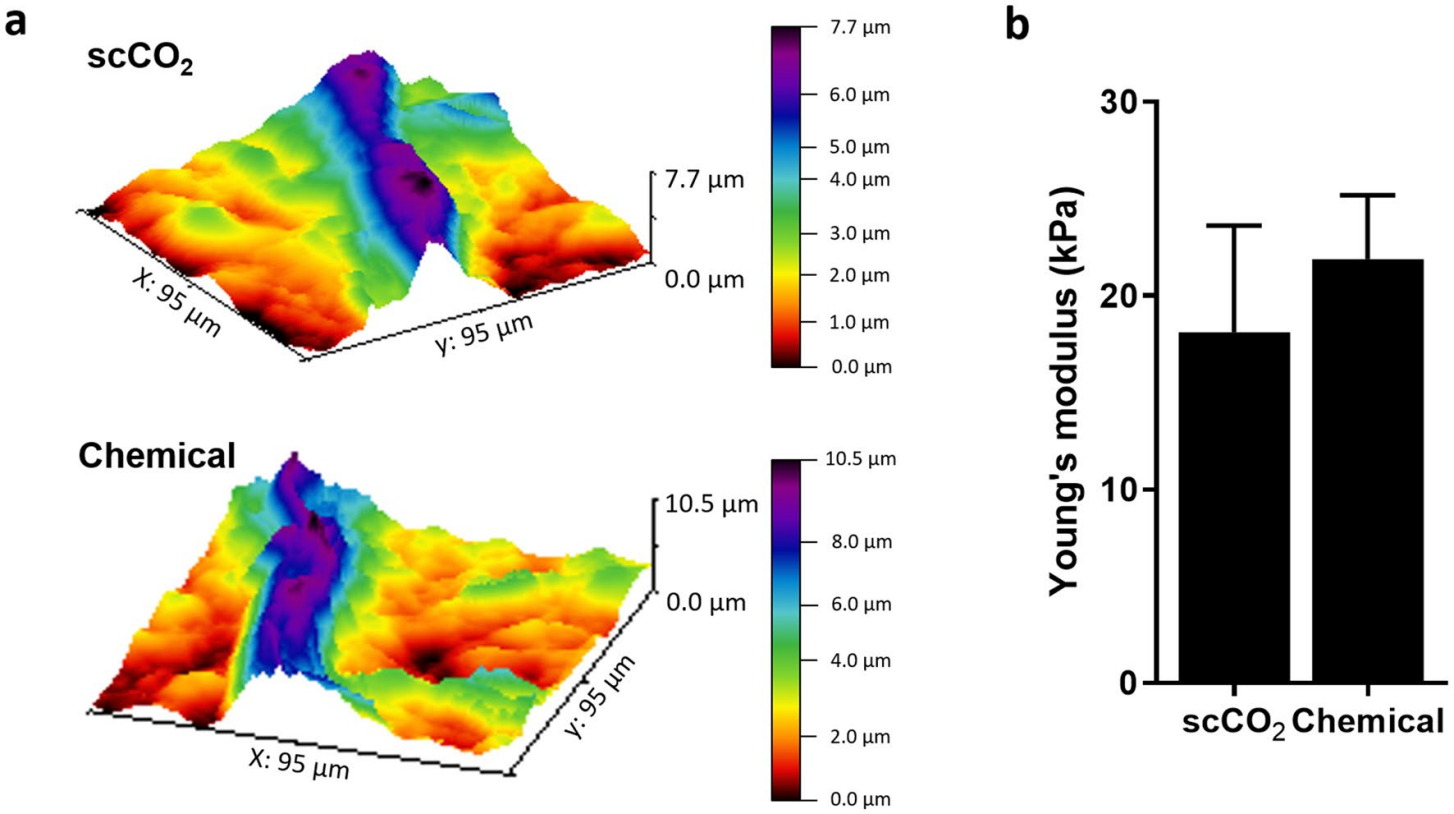
AFM imaging and spectrometry measurement. (**a**) Representative false colored three-dimensional surface mapping images and (**b**) Young’s modulus of scCO_2_ and chemically decellularized scaffolds (data as mean ± SEM; n = 5).

To determine if the scCO_2_ treatment could affect the mechanical properties of the decellularized tissue, we assessed the stiffness of the resulting scaffolds using Atomic Force Microscopy  (AFM). First, wide-area Z-scanning images revealed the heterogeneous surface topography of both plant scaffolds, with features ranging in height over 8 µm, yet confirming the preservation of structures such as plant vasculature (Fig. 3a). Next, force curves were acquired from a more homogeneous surface section (10 µm × 10 µm), avoiding scaffold features such as stoma or veins, to calculate the Young’s Modulus (YM). Although the difference was not significant, the YM of the scCO_2_ decellularized scaffold was found to be lower (18.08 kPa) than the chemically decellularized scaffold (21.88 kPa) (Fig. 3b).

### Scaffold composition by FTIR spectroscopy

**Figure 4:**
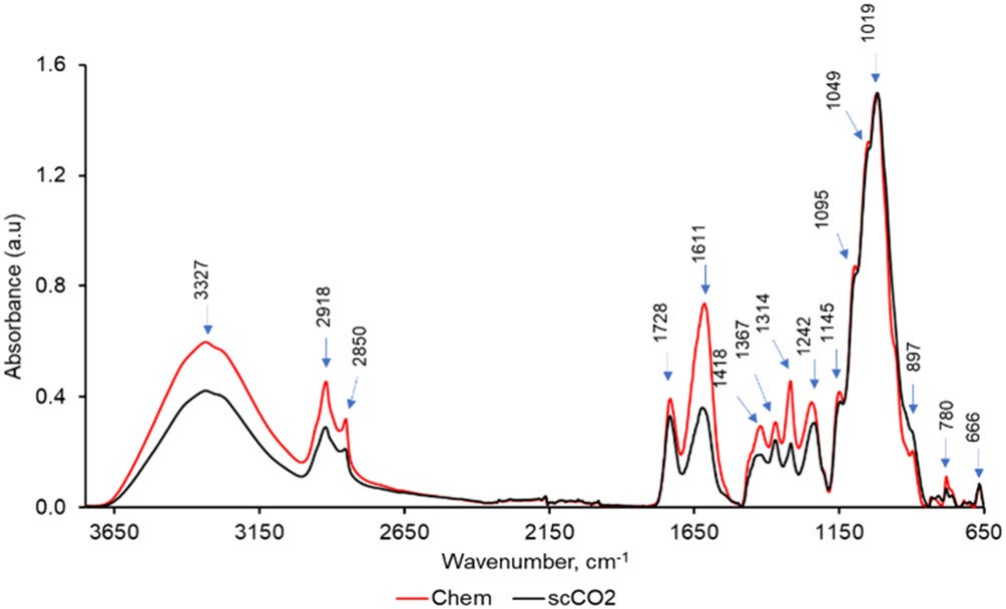
Baseline corrected and normalized Universal attenuated total reflectance - Fourier-transform infrared spectroscopy (UATR-FTIR) spectra acquired from chemically (Chem) and scCO_2_ decellularized baby spinach leaf scaffolds (n = 7).

FTIR spectroscopy provides information on the chemical composition in a wide range of materials. The infrared bands are assigned to different chemical functional groups present in a sample and the characteristics of infrared bands (such as location, intensity, area, and width) provide qualitative and quantitative information of biomolecules^44^. For example, the area and intensity of an infrared vibration indicates the relative abundance of that particular chemical functional group. Therefore, we assessed biochemical differences of chemical and scCO_2_ decellularized scaffolds using FTIR spectroscopy. Similar to many biological materials, leaf scaffolds produced complex and information-rich infrared spectra in the mid-IR region with many distinctive infrared vibrations, which were assigned to functional groups originating from macromolecules commonly found in plants, including cellulose, hemicellulose, lignin, pectin, and wax substances (Fig. 4). Table 1 summarizes the main infrared vibrations with their functional group assignment identified in scCO2 and chemically decellularized scaffolds.

**Table 1:** Position of infrared bands in chemically and scCO_2_ decellularized spinach leaf scaffolds with their functional group assignment.

Peak	Peak location (cm^−1^)		Assignment
	scCO_2_	Chemical	
1	3334	3335	Intra- and inter molecular hydrogen bonding^45^
2	2923	2921	CH_2_ anti-symmetrical stretching of wax substances with some contribution from other macromolecules such as carbohydrates^59,60^
3	2855	2853	CH_2_ symmetrical stretching of wax substances with some contribution from other macromolecules such as carbohydrates^59,60^
4	1734	1732	C = O stretch of esters^59^
5	1620	1614	Aromatic C = C stretch and/or asymmetric C-O stretch in COO- of lignin^59^ or asymmetric COO- of pectin^61^
6	1423	1422	CH_2_ scissoring of cellulose and also of hemicellulose, lignin, pectin^45^
7	1370	1369	C–H deformation of phenolic and aliphatic compounds (e.g. lignin)^59^
8	1317	1318	CH_2_ rocking vibration of cellulose^62^ and aliphatic materials present in the cuticle^60^
9	1241	1245	C–O–C stretching of esters^59^ (e.g. pectin) and OH stretching of cuticle substances^60^
10	1149	1149	C–O–C stretching of non-cellulosic materials such as hemicellulose and pectin^45^
11	1096	1095	Anti-symmetric in-plane stretching^45^
12	1048	1049	C-O stretch of cellulose, hemicellulose, and pectin^63^
13	1018	1019	C-O stretch of cellulose, hemicellulose, and pectin^63^
14	898	897	β linkage of cellulose^45^
15	780	779	C-H wagging that could originate from hemicellulose, pectin, and lignin^64^
16	666	666	OH out-of-plane bending that could originate from cellulose, hemicellulose, and lignin^45^

**Figure 5:**
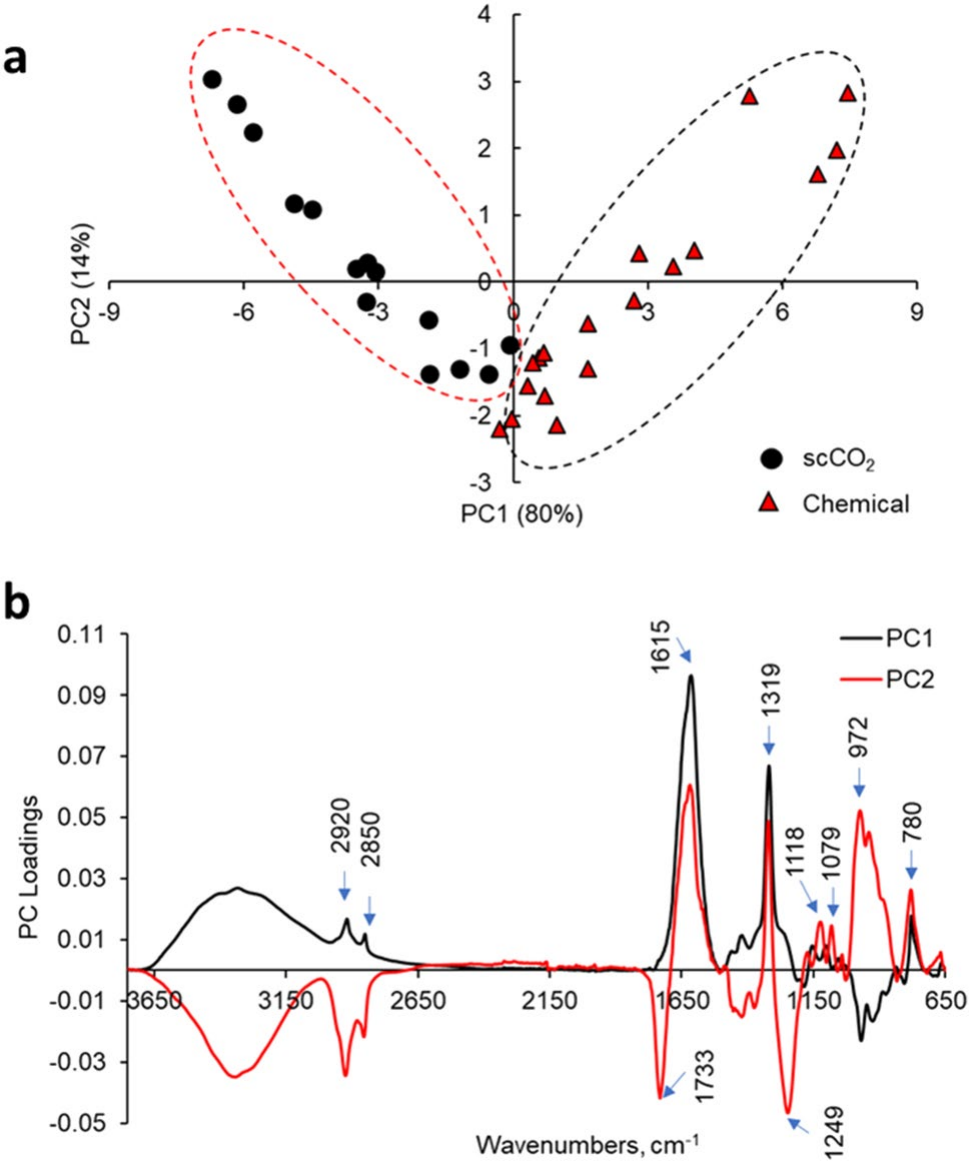
PCA analysis of UATR-FTIR spectra acquired from chemically and scCO_2_ decellularized baby spinach leaves. (**a**) PCA score plot where each data point represents an individual spectrum. (**b**) PC1 and PC2 loadings as a function of wavenumbers (n = 7).

Figure 4 shows typical FTIR spectra acquired from chemically and scCO_2_ processed leaf scaffolds. These spectra appeared to be similar. However, visual comparison of the spectra from each scaffold showed differences in peak intensities due to the relative quantity of the remaining biomolecules in each type of scaffold. To analyze these spectra and determine critical spectral differences between chemically and sCO_2_ decellularized leaves, we applied a multivariate data analysis technique. Principal Component Analysis (PCA) is widely used to reduce the dimensionality of spectroscopic data and group the FTIR data based on spectral similarities^45^. Figure 5a shows the PCA of the FTIR spectra of chemically and scCO_2_ decellularized spinach leaves. PC1 accounts for 80% of the total variance and separates the spectra of chemically decellularized scaffolds from those of scCO_2_ decellularized, while PC2 accounts for 14% of the total variance. A large variability within each group was noticed, which may indicate that spinach leaves were not decellularized to the same level or due to within plant and plant-to-plant biochemical differences in fresh leaves (age of the plant, location of the leaf, etc.). Furthermore, the separation of the spectra into two groups according to the method of decellularization indicated that chemical decellularization and scCO_2_ decellularization were not impacting the biochemical composition of scaffolds the same way.

The plots of PC1 and PC2 loadings as a function of wavenumbers are shown in Fig. 5b. The vibrations 2920, 2850, 1615, 1319, and 780 cm^−1^ contributed most to spectral separation along PC1 axis. These vibrations originated from cellulose, hemicellulose, pectin, lignin, and wax substances. Based on this result and histochemical investigations with ruthenium red (for pectin staining), calcofluor white (for cellulose staining), toluidine blue (for lignin/pectin staining), and oil red O (for wax staining) (data not shown), chemical and scCO_2_ methods appeared to remove some biomolecules even from the extra cellular matrix of leaves and, thus, the chemical profiles of decellularized scaffolds appeared slightly different from each other. Such compound may include pectin and wax substances that were easily removed during the decellularization process compared to that of more stable molecules such as cellulose and lignin.

### Scaffold elemental distribution

**Figure 6:**
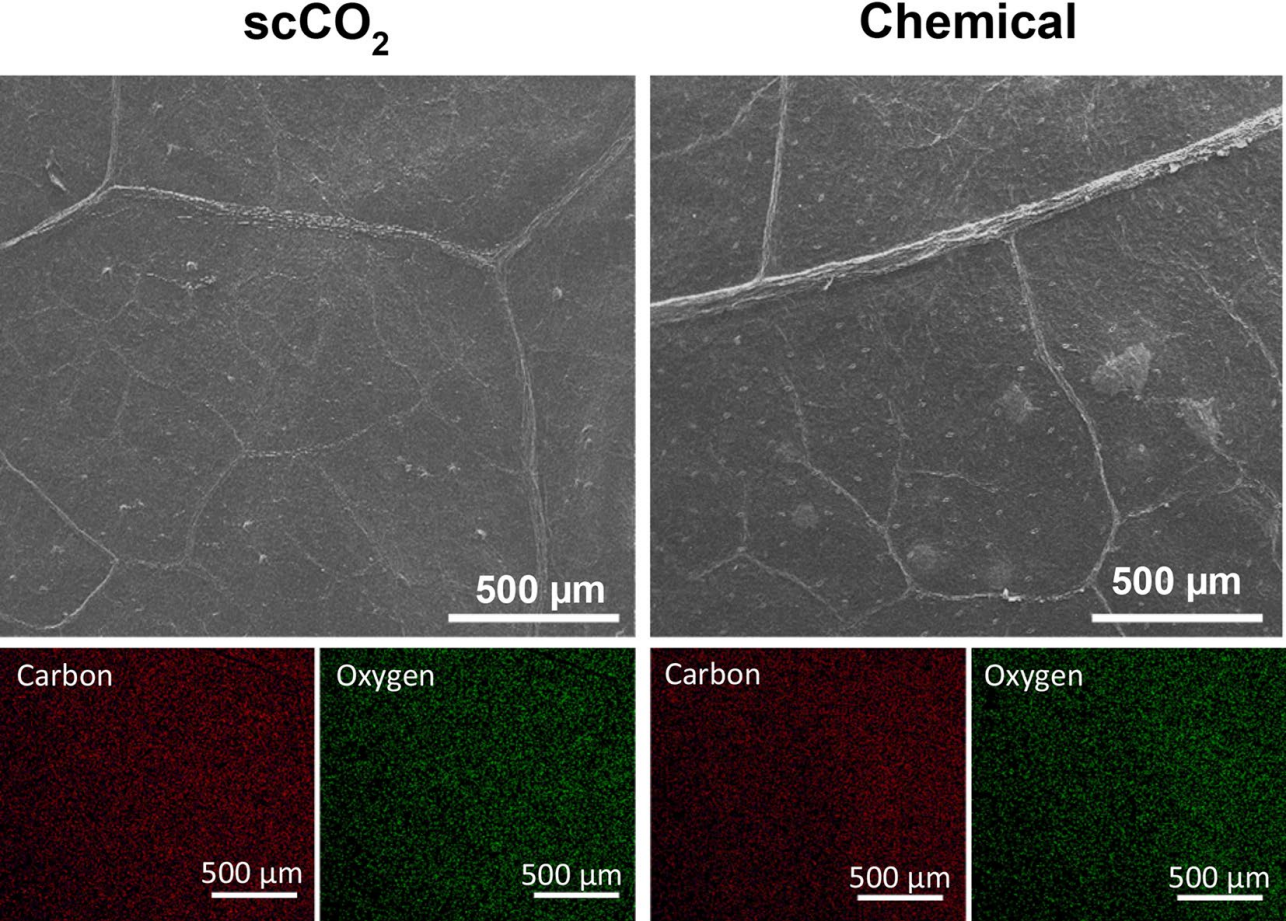
Elemental analysis mapping of scCO_2_ and chemically decellularized scaffolds. SEM images (above) and corresponding elemental maps (below) for the most abundant elements, carbon (red) and oxygen (green).

To complement the FTIR investigation, the elemental composition of the two scaffolds was evaluated and summarized in Table 2. The distribution between the two scaffolds was similar. Carbon (Fig. 6, red) and oxygen (Fig. 6, green) were the most abundant elements, averaging 56% and 40% respectively. These two elements, that comprise the most common plant macromolecules such as cellulose, lignin, pectin and hemicellulose, as expected, were found throughout the plant scaffold, including plant tissue and vascular networks (Fig. 6). The remaining elements composed only 3% of the scaffold. Sodium was the third most present element (approximately 2%) while sulfur displayed the lowest presence in both scaffolds (0.05%).

**Table 2:** Mass percentage (mean ± SD) of the elements present in the decellularized spinach scaffolds.

Element	Symbol	scCO_2_ (%)	Chemical (%)
Carbon	C	57.77 ± 1.96	55.65 ± 1.33
Oxygen	O	39.06 ± 2.39	41.61 ± 0.06
Sodium	Na	1.87 ± 0.18	2.01 ± 0.24
Magnesium	Mg	0.05 ± 0.04	0.08 ± 0.04
Silicone	Si	0.07 ± 0.04	0.06 ± 0.05
Phosphorus	P	0.30 ± 0.12	0.03 ± 0.03
Sulfur	S	0.05 ± 0.04	0.05 ± 0.03
Chlorine	Cl	0.61 ± 0.18	0.69 ± 0.21
Potassium	K	0.23 ± 0.03	0.15 ± 0.03

### Biocompatibility of scCO_2_ decellularized scaffold

**Figure 7:**
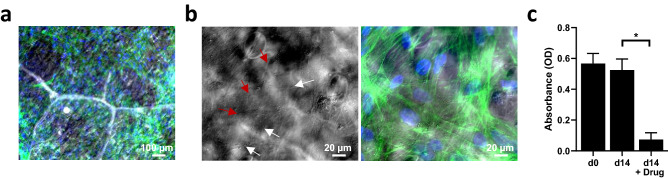
Human dermal fibroblast cell adhesion and viability on scCO_2_ decellularized scaffold. (**a**) Low magnification image merging scCO_2_ decellularized scaffold phase contrast picture with IF picture of BJ cells stained for F-actin (green) and DAPI (blue). (**b**) Close-up images showing phase contrast picture (left) of the leaf scaffold revealing the imprints of the cell nucleus (red arrow) and clusters of cell attachment points (white arrows) merged with IF picture of BJ cells (right) stained for F-actin (green) and DAPI (blue). (**c**) Cell viability of BJ cells on scCO_2_ decellularized scaffold, assessed by MTT immediately after seeding (d0) and after 14 days of culture (d14), in presence or not of 0.04 µg/ml puromycin (Drug). p < 0.0057 (data as mean ± SEM; n = 3).

In order to assess scCO_2_ decellularized scaffold compatibility with human cells, scaffolds were seeded with human normal dermal fibroblast (BJ) cells. After 3 days, BJ cells were found across the scaffold surface and displayed long-shape architecture, suggesting that cells can effectively attach to the scCO_2_ decellularized scaffolds (Fig. 7a). The shape of the nuclei can be seen imprinted on the leaf scaffold (red arrows) while the point of cell attachment to the scaffold can be seen with small, clustered imprints (white arrows) (Fig. 7b).

As cell attachment was confirmed, cell viability was then evaluated using a modified MTT. Results revealed that after 14 days, the cells were alive on the scCO_2_ decellularized scaffold while the viability decreased in response to 0.04 µg/ml of puromycin drug exposure (Fig. 7c). Together, these results indicate that similar to the chemically decellularized scaffolds, normal human cells can attach, survive and respond to a drug stimulus on the scCO_2_ decellularized plant scaffold.

## Discussion

**Figure 8:**
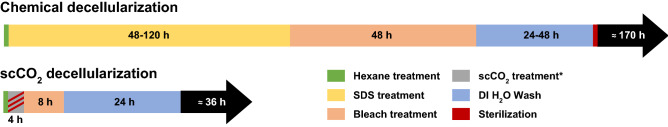
Time comparison of the scCO_2_ and traditional chemical decellularization techniques. *device preparation and decompression time included.

ScCO_2_ has broad applications including extraction in petrochemistry, separation technology in food industry to obtain natural compounds of high quality, fluid extraction of biomass constituents, green materials processing and synthesis (3D aerogel or coating) or extrusion processes^46,47^. In this study, we showed that scCO_2_ is also a successful approach to decellularize plant material. Using moderate pressure and a PAA entrainer, the dissolving power of the fluid effectively removed plant cellular content to generate a biocompatible scaffold in mere hours. The gold standard chemical processing of plant material decellularization is time-consuming, taking several days (Table S1)^6,8,9,11,12,14^. Leaves are mechanically agitated in a bath of SDS/detergent for anywhere from 2 to 5 days. This process is followed by 1–2 additional days of decoloring and sterilization in hypochlorite solution and followed by subsequent washes with DI water. Additional freeze-drying or sterilization steps can also be performed. scCO_2_ processing streamlines this lengthy process and achieves the same result in less than 1/5 the time on average (Fig. 8).

Further, plant scaffolds must be sterilized before introduction to cell culture environments. Sodium hypochlorite, UV radiation or ethanol methods have been previously employed. While successful, scCO_2_ has an established history of use for sterilization in the food, pharmaceutical and biomedical industries^42,48,49^. Recently, scCO_2_ sterilization has been demonstrated on various human tissues for tissue grafting such as lung matrices, amniotic membranes, tendon and bone as well as heart valves^36,38,50,51^. Similarly, PAA has long been used to disinfect medical equipment^52^. Its low molecular weight allows PAA to rapidly dissolve into the compressed CO_2_ and enhance the solvent power^53^. PAA rapidly degrades into acetic acid and water which eliminates the formation of a toxic residue. Interestingly, scCO_2_ with PAA additives has also been shown to synergistically inactive bacterial spores^42,54^. Therefore, our approach using scCO_2_ + PAA may have the unique ability to simultaneously perform decellularization with sterilization. To demonstrate this capability on plant scaffolds, we aseptically opened our tubes containing scCO_2_ processed leaf scaffolds and incubated them in various bacterial broths. After 72 h, bacterial growth was not observed (Figure S3) compared to a fresh leaf which displayed high turbidity. While scCO_2_ decellularization/sterilization of plant tissue should be validated using a variety of microorganisms, this technique shows great promise for one-step decellularization/sterilization.

The hypothesis that scCO_2_ achieves decellularization by high pressure bursting of cell membranes is not widely accepted and has been largely disproven^42,48,55^. Instead, pressure allows CO_2_ to dissolve into the liquid phase and facilitates the conditions for the fluid to penetrate the plant cell wall, through the cell membrane and, once inside of the cell, act as a powerful solvent. This mass transfer of scCO_2_ lowers the intracellular pH by the formation of carbonic acid, enhanced in our case by the presence of PAA, leading to the disruption of cell metabolism and the removal of essential enzymes without compromising the overall scaffold structure. Evidence for this mechanism of action is seen in the preserved plant cell architecture (Fig. 2) where intact cell walls and vascular networks of scCO_2_ scaffolds were visualized. Due to some leakage, the scCO_2_ vascular preservation appears to be superior to the chemical scaffold. While this may vary from leaf to leaf, recall that ionic detergents like SDS are known to degrade proteins and damage scaffold ultrastructure. However, our AFM study of the microscale stiffness found that the scCO_2_ processed scaffolds were less stiff than the chemically processed scaffolds. While this difference was not significant, AFM technology only represents nanomechanical measurements, and bulk mechanical properties of the scCO_2_ decellularized scaffolds must also be investigated. With a limited dataset, we performed preliminary bulk tensile measurements and found that the tangent modulus and the ultimate tensile strength of scCO_2_ decellularized scaffolds were lower than chemically treated scaffolds (data not shown). While these results require further validation, this may suggest that decellularized scaffolds are indeed softer after scCO_2_ treatment and could be difficult to manipulate. However, we also hypothesize that this may vary from plant to plant and the decellularization method should be carefully selected according to the type of material, viscoelastic properties, desired use and measured outcome.

In addition, UATR-FTIR was a highly sensitive technique used to demonstrate the biochemical differences between decellularized scaffolds. As expected, results showed the presence of common plant macromolecules in both scaffolds such as cellulose, hemicellulose, lignin or pectin, yet the presence of wax substances was unexpected. We hypothesized the outer wax cuticle was removed during decellularization. Moreover, within each group, a high variability was found. The source of such variability could be from the decellularization process or the result of plant variation. Indeed, plants display high inter- and intra-variation stemming from both genetic and environmental conditions^56^. Further investigation is needed to understand the effects of these differences on the behavior of repopulated mammalian cells.

Finally, human dermal fibroblast cells have been seeded on scCO_2_ decellularized scaffolds and our results found that they were attached, viable at 14 days and able to respond to a drug stimulus. Although we did not assess scaffold immunogenicity in this study, these results suggest that scCO_2_ decellularization of vegetal material is indeed a non-toxic approach to provide biocompatible scaffolds for human cell culture. Several types of mammalian cells have been cultured on chemically decellularized vegetal scaffolds, including cancer cell lines, mesenchymal stem cells, mouse fibroblasts, human-induced pluripotent stem cell-derived cardiomyocytes, and more^6,8,9,11,12^. Although we foresee that scCO_2_ decellularized scaffolds could also accommodate a diversity of cell types, additional investigations are required to confirm this assumption.

## Summary

ScCO_2_ is an efficient method to decellularize plant material, faster than the gold standard chemical approach, which allows the resulting scaffolds to be sterile and to support the culture of human cells. Our biochemical and biophysical analysis displayed minor differences between the two treatments, and further investigation would be required to assess if these changes are significant and impact behavior of repopulated cells. While we report the success of decellularized vegetal material with scCO_2_ and PAA co-solvent, it is likely that each plant will require minor adjustments to this protocol to facilitate optimal decellularization. The most effective parameters will depend upon a plant tissue’s cellular density, lipid content, xylem configuration, leaf thickness, or wax cuticle^30^.

## Material and methods

### Chemical and scCO_2_ decellularization

Baby spinach leaves (*Spinacia oleracea*), sweet mint plants (*Mentha x suavis*), celery stalks (*Apium graveoleans*) and curly parsley (*Petroselinum crispum*) were purchased from the local store. The wax cuticle was removed by alternating washes of hexane and phosphate buffered saline solution (PBS).

For scCO_2_ decellularization, individual leaves were placed in a vented 15 mL conical tube with 5 mL of co-solvent. The lids were fastened onto the tubes yet left loose to allow more opportunity for the fluid to penetrate the tube. A maximum of three tubes were placed into the 600 mL pressure vessel of a NovaGenesis500 (Novasterilis, NY, USA) which was sealed with 33.9 Nm of torque. Liquid CO_2_ (0.56 kg/run, medical grade (99.9% purity), syphon tube) flowed into the vessel and the pressure was increased using a high-pressure CO_2_ pneumatic booster pump (0.55 MPa at 3.2^–5^ cubic meters/cycle). The pump was cycled approximately 5 times to meet the desired condition of 17.23 MPa and 33 °C. Time clock was initiated when temperature and pressure equalized and ran for 180 min. During scCO_2_ exposure, the pressure was held constant at 17.23 MPa and a heater was used to maintain the temperature at 33 °C inside of the pressure vessel as measured by an inner-vessel type J thermocouple. After the desired exposure time, the pressure vessel was depressurized using a manual, 3-way ball valve in addition to a CO_2_ empty metering valve at a rate of 0.69–1.38 MPa/min to maintain temperature as well as to limit potential scaffold damage. Leaves were then removed from the pressure vessel, aseptically opened in a sterile tissue culture hood, flushed with bleach for 8 h and washed with deionized (DI) water for 24 h. The device’s external O-rings and CO_2_ filters were replaced every 45 runs. The pressure relief valve was replaced every six months. The machine was annually inspected and maintained by manufacturer NovaSterilis.

For chemical decellularization, spinach leaves were first cannulated through the petiole (base of the stem) with a 26-gauge needle and secured to the leaf stem with heat shrink tubing. The prepared leaves were connected to gravity bags which were filled with various solutions used for decellularization, starting with 1% sodium dodecyl sulfate (SDS) in DI water for 48 h. This was followed by a solution of 10% sodium chlorite and 0.1% Triton-X 100 in DI water for 48 h. The leaves were then flushed with DI water for an additional day.

### DNA and protein quantification

Leaf material (25 mg) was flash-frozen using liquid nitrogen and ground by mortar and pestle as previously reported^11^. Resulting material was transferred to a microcentrifuge tube for DNA and protein quantification. DNA content was extracted using DNeasy Plant Mini Kit (Qiagen) following manufacturer recommendation, while proteins were extracted by radioimmunoprecipitation assay (RIPA) (Pierce RIPA buffer, ThermoFisher Scientific) for 30 min on ice. DNA content was quantified by reading the absorbance at 260 nm and the protein content was quantified using MicroBCA protein assay kit (ThermoFisher Scientific). Both absorbances were measured using Epoch microplate spectrophotometer (BioTek Instruments).

### Scanning electron microscopy (SEM) and energy-dispersive X-ray spectroscopy (EDS)

Decellularized scaffolds were fixed with 2.5% glutaraldehyde in PBS overnight at 4 °C. Samples were then dehydrated in increasing concentrations of ethanol (50, 70, 85, 95, 95, 100, 100%) for one hour each and left overnight in 100% ethanol at 4 °C. Samples were mounted on pin stubs (12.7 mm × 8 mm, Ted Pella) and sputter coated with gold (10 nm) under vacuum for direct SEM imaging or with carbon for EDS. Samples were imaged at the Eyring Materials Center at Arizona State University with a SEM-FEG XL30 (FEI) and EDS data acquired using Genesis Spectrum software version 5.21. (EDAX Inc., New Jersey, USA https://www.edax.com/).

### Water retention quantification

Plant scaffold tissue was dried for 48 h at room temperature and the weight of the dried tissue was recorded. Next, the plant tissue was immersed in 2 mL of DI water for 1 h at room temperature and weighed a second time after rehydration. The weight difference was considered as the amount of water retained by the tissue.

### Assessment of scCO_2_sterilization efficiency

Scaffolds were removed from the pressure vessel, aseptically opened in a sterile tissue culture hood and washed with DI water for 24 h. Leaves were cut in half and placed in Erlenmeyer flasks. Each flask was filled with 25 mL of one of four different bacterial broths, Tryptic Soy (BD), Nutrient Broth (BD), Brain Heart Broth (Sigma) or Lactobacilli MRS (BD). Turbidity was measured after 72 h incubation under shaking at 35 °C by a UV/VIS Spectrophotometer (Unico, USA) at 600 nm wavelength.

### Atomic force microscopy (AFM)

Samples were first imaged on a large area (95 µm × 95 µm) using a Nanosurf Flex-Bio AFM System (Nanosurf, Switzerland) before determining the Young’s Modulus (YM) on a more homogenous section (10 µm × 10 µm) using force spectroscopy mode at liquid interface (Figure S2). Color-gradient topography maps (Fig. 3a) extracted from imaging were generated using Gwyddion software version 2.56 (SourceForge, http://gwyddion.net/). Gold coated qp-BioAC cantilevers (Nanosensors), 80 μm in length, 30 μm in width, 400 nm thick, with a nominal force constant of 0.06 N/m and a resonance frequency of 30 kHz were used for scaffold measurement. The samples were first fixed to a glass slide with vacuum grease and mounted on a magnetic AFM stage at room temperature (24–26 °C). Next, the spring constant of the cantilever was calibrated by using the thermal tune method on a cleaned and stiff surface (Mica) and then force curves were measured. For each plant, force maps were recorded at 3 different positions on the same leaf for at least 5 different leaves. Each force map contained 64 force curves (8 × 8 lines per frame) (Figure S2) and were processed with the C3000 Nanosurf software version 3.10.0 (https://www.nanosurf.com/en/software/c3000). The YM was calculated with AtomicJ 1.7.2 software^57^, using the Harding and Sneddon model^58^ and the equation $P= \frac{2E Tan [\theta ]}{\pi (1-{\nu }^{2})} {\delta }^{2}$ where P is the load force, E is the YM, θ is the half-angle (15°), ν is the Poisson’s ratio (0.5) and δ is the depth of indentation.

### Fourier-transform infrared (FTIR) spectroscopy

Decellularized leaf scaffolds were dehydrated at 50 °C for 5 h and ground to a fine powder using a laboratory mortar and pestle. FTIR spectra of leaf scaffold powder were acquired using Spectrum-400 spectrometer equipped with a universal attenuated total reflectance (UATR) accessory (PerkinElmer, USA). Samples were directly deposited on ZnSe crystal and a constant pressure was applied to ensure a good contact between the sample and the infrared beam. At least two spectra were acquired from each scaffold in the mid IR range (4000–650 cm^-1^) with 4 cm^-1^ spectral resolution and 32 co-added scans. A background spectrum of the clean ZnSe crystal was recorded prior to data collection. The crystal was cleaned with Milli-Q water and properly dried before recording each spectrum. Spectra normalization and baseline correction were performed using Spectrum 10 software (PerkinElmer, USA). The preprocessed spectra were exported to Unscrambler X software version 9.6 (CAMO Software AS, Norway, https://www.camo.com/unscrambler/) and principal component analysis (PCA) was performed.

### Assessment of vascular integrity

Capillary tubing (0.5 mm inner-diameter, 1 cm in length) was inserted into the petiole of a fresh leaf before decellularization processing as previously described. Upon completion of decellularization, the capillary tubing, which remained in the base of the stem, was placed into a reservoir of ponceau red dye. The scaffold was supported at a 45-degree angle to the benchtop and was allowed to dry overnight at room temperature before images were acquired with a standard camera. As the moisture in the leaf evaporated, the fluid was drawn up into the capillary tubing and throughout the vascular network, similar to a tree’s water transport by evaporation driven pump^43^.

### Cell seeding and immunofluorescence (IF)

Human dermal fibroblast cells (BJ, CRL-2522, ATCC) were cultured in Eagle’s Minimum Essential Medium, supplemented with 10% fetal bovine serum and 1% Penicillin/Streptomycin and maintained at 37 °C in 5% CO_2_ atmosphere. All leaf structures were functionalized with collagen and fibronectin proteins as previously described^11^. Briefly, scaffolds were incubated in 50 ug/ml of collagen I (A1048301, Thermo Scientific) in 20 mM acetic acid solution for 4 h, followed by two washes in PBS and a final wash in complete medium. Leaves were then incubated in 10 ug/mL fibronectin (F0895, Sigma-Aldrich) for 24 h followed by three washes in complete medium.

Cells were seeded at 6,000 cells/cm^2^ on functionalized leaves. As previously reported, after 72 h, cells were fixed with 4% paraformaldehyde in PBS for 15 min and permeabilized with 0.1% Triton X-100/PBS for 5 min^11^. Then, cells were blocked for 30 min at room temperature with 1% bovin serum albumin in PBS and immunostained with Alexa Fluo 647-conjugated Phalloidin (#A22287, Life Technologies, 1/40) and counterstained with 4′,6-diamidino-2-phenylindole (DAPI). Images were obtained using a Zeiss Axio Imager M2 epifluorescent microscope and were acquired with a Zeiss AxioCam MRm camera using ZEN 4.5 software at the Biomedical Imaging Core Facility at the UA College of Medicine – Phoenix (Zeiss, https://www.zeiss.com/microscopy/us/products/microscope-software/zen.html).

### Cell viability assay

A modified MTT experiment was previously developed from CellTiter 96 NonRadioactive Cell Proliferation Assay kit (Promega)^11^. Treated leaves were cut into pieces to fit an untreated 96-well plate. Cells were seeded at 6,000 cells/cm^2^. For drug response, puromycin was added at a concentration of 0.04 µg/ml. The medium was refreshed every 72 h. To avoid measuring any residual cells not attached to the scaffold, leaves with attached cells were transferred to a new plate. Tetrazolium component was added at day 0 and 14 and the absorbance of formazan product was measured at 570 nm by using Epoch microplate spectrophotometer (Biotek Instruments).

### Statistical analysis

All statistical tests and graphs were performed with GraphPad Prism version 8.00 for Windows (GraphPad Software Inc., La Jolla, CA, https://www.graphpad.com/scientific-software/prism/). All results are presented as mean ± SEM or mean ± SD using a Student’s t-test. Statistical results were considered significant for p < 0.05.

## Supplementary Information

Supplementary Information 1.
