# Supplementary material for: Supercritical carbon dioxide decellularization of plant material to generate 3D biocompatible scaffolds
Source: Sci Rep. 2021 Feb 11;11:3643. doi: 10.1038/s41598-021-83250-9 (PMC7878742; doi:10.1038/s41598-021-83250-9)
Supplement: Supplementary file 1 — Supplementary Information 1. [file 41598_2021_83250_MOESM1_ESM.pdf]

Supercritical carbon dioxide decellularization of plant material to generate 3D biocompatible scaffolds

Ashlee F. Harris[1]†*, Jerome Lacombe[1,2]†*, Sumedha Liyanage[3], Margaret Y. Han[1], Emily Wallace[1], Sophia Karsunky[4], Noureddine Abidi[3] and Frederic Zenhausern[1,2,4*].

[1] Center for Applied NanoBioscience and Medicine, College of Medicine Phoenix, University of Arizona, 475 North 5th Street, Phoenix, Arizona, USA 85004
[2] Department of Basic Medical Sciences; College of Medicine Phoenix, University of Arizona, 475 N 5th St., Phoenix, Arizona, USA 85004
[3] Fiber and Biopolymer Research Institute, Department of Plant and Soil Science, Texas Tech University, Lubbock, Texas, USA
[4] School of Pharmaceutical Sciences, University of Geneva, Geneva, Switzerland

†These authors contributed equally to this work

Supplementary Material

Figure S1

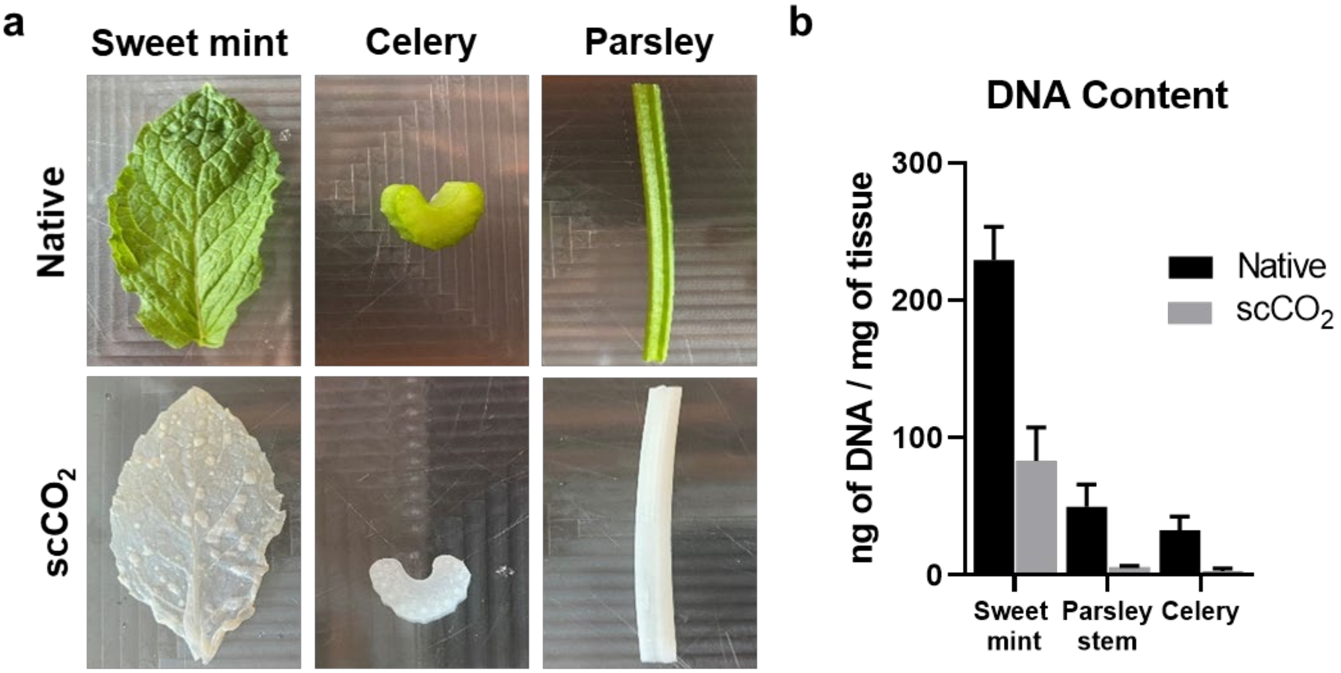

Figure S1. $scCO_2$ decellularization of additional plant material. a) Images of mint leaves, celery stalks and parsley stems before (native) and after $scCO_2$ decellularization treatment. b) DNA quantification of native and $scCO_2$ decellularized mint leaves, celery stalks and parsley stems (data as mean ± SEM; n=3).

Figure S2

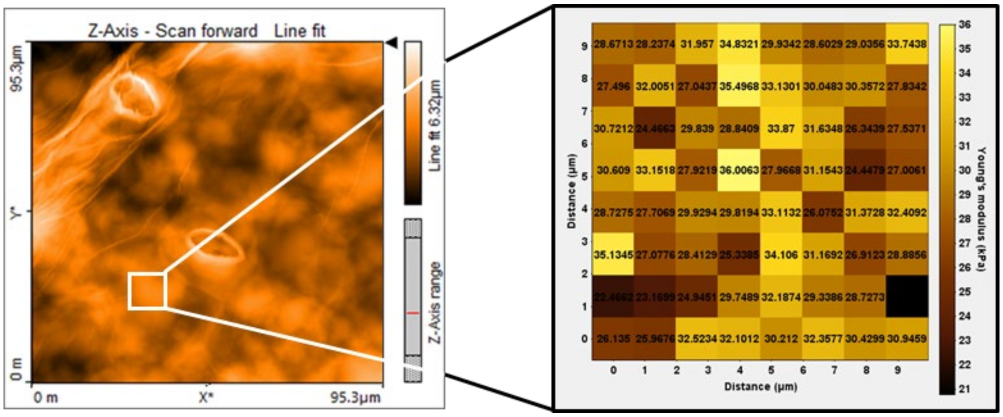

Figure S2. Representative Z-scan image of decellularized plant material. Inset is a 10 x 10 µm area from which force curves are generated. The histogram is representative of the Young's modulus data generated from these areas.

Figure S3

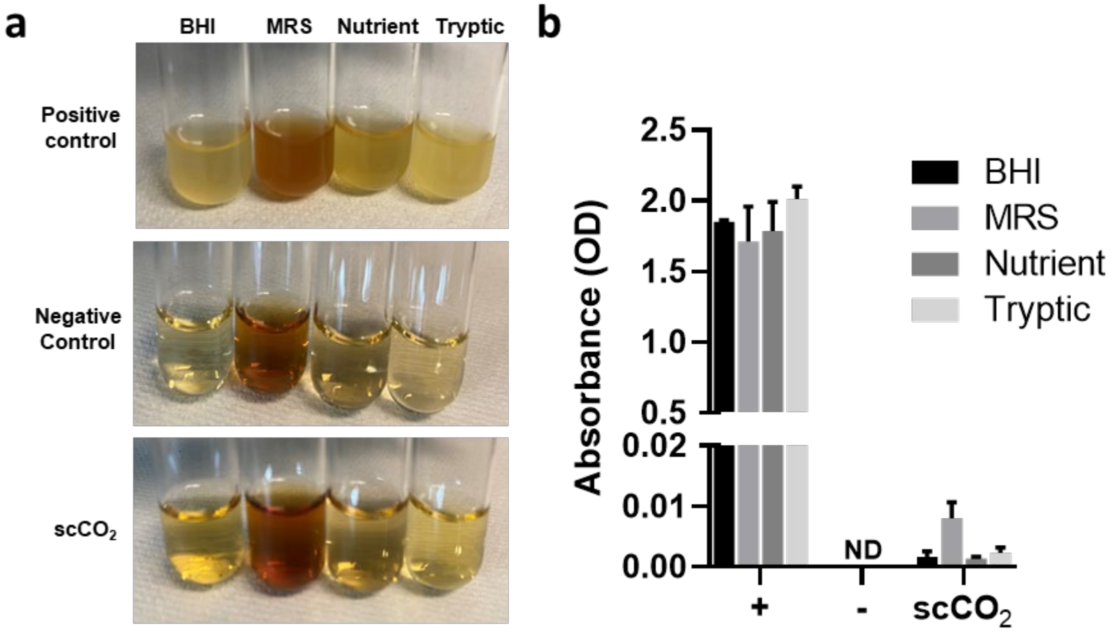

Figure S3. Assessment of $scCO_2$ technique for scaffold sterilization. a) Images of four bacterial broths' turbidity after 72 hours incubation with fresh spinach leaf (positive control), broth alone (negative control) and $scCO_2$ treated scaffold. b) Quantification of broth turbidity (data as mean ± SEM; n=3). $scCO_2$ measured absorbance could be caused by the presence of scaffold debris.

Table S1
Time of spinach leaf decellularization using standard chemical treatment (in hours)

	Gersklak, *et al.* (8)	Fontana, *et al.* (9)	Dikici, *et al.* (10)	Robbins, *et al.* (12)	Lacombe, *et al.* (11)	Salehi, *et al.* (14)	Wang, *et al.* (16)
Hexane Treatment	0.5*	0.5*	1	0.5*	0.5*	0.5	0.25
SDS/Detergent	120	120	120	24	48	120	24
Bleach/Surfactant	48	48	28	24	48	48	24
Washing	48	48	2	36	48	0.5*	24
Sterilization			48	0.5	0.5		2
Freeze-dried						72	
**Total**	**216**	**216**	**199**	**85**	**145**	**240**	**74.25**

*As treatment/wash times were undefined, 3 cycles were equated to 0.5 hour
